# Revolutionizing Senescence Detection: Advancements from Traditional Methods to Cutting-Edge Techniques

**DOI:** 10.14336/AD.202.0565

**Published:** 2025-07-01

**Authors:** Ruopeng Xiao, Sinan Hu, Xiaohui Du, Yiwen Wang, Ke Fang, Yibin Zhu, Nanbin Lou, Chunhui Yuan, Jing Yang

**Affiliations:** Department of Basic Medicine, School of Medicine, Hangzhou City University, Hangzhou, Zhejiang, China

**Keywords:** senescent cells, aging, biomarkers, detection methods

## Abstract

The accumulation of senescent cells is an important factor in the complex progression of aging, with significant implications for the development of numerous diseases. Thus, understanding the fundamental mechanisms of senescence is paramount for advancing preventive and therapeutic approaches to age-related conditions. Important to this pursuit is the precise identification and examination of senescent cells, contingent upon the recognition of specific biomarkers. Historically, detection methods relied on assessing molecular protein and mRNA levels and various staining techniques. While these conventional approaches have contributed substantially to the field, they possess limitations in capturing the dynamic evolution of cellular aging in real time. The emergence of novel technologies has led to a paradigm shift in senescence research. Gene-edited mouse models and the application of advanced probes have revolutionized our ability to detect senescent cells. These cutting-edge methodologies provide a more detailed and accurate means of dynamically monitoring, characterizing and potentially eliminating senescent cells, thus enhancing our understanding of the complex mechanisms of aging. This review comprehensively explores both traditional and innovative senescent cell detection methods, elucidating their advantages, limitations and implications for future investigations and could serve as a comprehensive guide and catalyst for further advancements in the understanding of aging and associated pathologies.

## 1. Introduction

Cellular senescence is an important biomarker of organismal aging and is characterized by stable cell cycle arrest and a distinct secretory profile adopted by cells in response to injury or stress [1-4]. This process aids embryonic development and wound healing. However, it also paradoxically contributes to the development of age-related diseases including degenerative conditions and cancers, thereby exacerbating the aging process overall. Given the expanding aging population and the extension of human lifespan, it is more critical than ever to study the mechanisms of cellular senescence. Understanding these mechanisms is vital not only for gaining a deeper insight into the aging process and the progression of diseases but also for identifying potential therapeutic targets. This knowledge could pave the way for developing strategies aimed at mitigating the effects of aging and improving health outcomes in the elderly.

In recent times, the most crucial approach to understanding the mechanisms behind cellular senescence involves the identification of biomarkers specific to senescent cells. However, due to the diversity and complexity of cellular senescence across organisms, its detection has consistently posed a challenge in scientific research. A case in point is the Senescence-Associated Secretory Phenotype (SASP) database, which illustrates the vast array of distinct secretory profiles within the SASP category. Furthermore, the composition of SASP varies significantly among different tissues, and certain protein characteristics change as cells age [5]. Variations in the components of SASP not only underscore the multifaceted nature of cellular aging but also highlight the pivotal role that tissue-specific SASP plays in regulating cellular interactions and the microenvironment. Traditional methodologies, such as measuring β-galactosidase activity, Western blotting (WB), quantitative PCR (qPCR) and morphological assessment following staining and antibody incubation, offer some level of senescent cell identification. Nevertheless, these methods are often cumbersome, prone to operational and environmental influences, and lack adaptability. Thus, there is an urgent need to develop new detection methods that improve the accuracy and precision of assessing cellular senescence.

Recent advancements in molecular biology have led to the development of a range of innovative biomarkers and detection techniques, which have improved flexibility and hold promise for application across cell cultures, tissue samples, and in vivo studies. For instance, various fluorescent proteins have been utilized for in vivo imaging of mice [6], and new near-infrared ratio fluorescent probes enable precise monitoring of key biomarkers during cellular aging [7]. These innovative methods not only enhance our ability to monitor cellular senescence but also provide a platform integrating senescence detection with drug development efforts. Many of these methods allow for in vivo detection, providing real-time insights and expanding our ability to track senescent cells. This review outlined an array of detection strategies for cellular senescence in mice, encompassing both established and emerging techniques such as gene-edited mouse models and sophisticated probes. We also discussed the underlying principles, strengths and limitations of each method and their suitability for various research contexts. Lastly, we provided a forward-looking perspective, exploring potential directions, challenges, and opportunities to optimize these strategies for advancing research and practical applications in the domain of cellular senescence.

## 2. Mechanisms and hallmarks of cellular senescence

Cellular aging, also known as senescence, is a complex biological process that ultimately leads to the cessation of cell division. This concept was first introduced by Leonard Hayflick in 1961. He observed that fetal fibroblasts ceased to divide after approximately 50 passages in vitro, even under optimal growth conditions [8]. Hayflick attributed this degenerative phenomenon to internal factors, using the term "senescence" to describe it. This observation led to the identification of an intrinsic limitation to cell proliferation, known as the Hayflick limit. Cells are the fundamental units of individual life, and the aging of an organism is believed to result from the accumulation of senescent cells [9-11]. In 2019, the International Cellular Senescence Association (ICSA) provided a standardized definition of cellular senescence. It is described as a state resulting from stress or specific physiological processes and is characterized by stable growth arrest, SASP, macromolecular damage such as DNA damage, and metabolic dysfunction [12].

Cellular senescence can be triggered by various internal and external stimuli. Telomere attrition serves as a primary trigger for senescence, where telomeres, with their TTAGGG sequence, shorten with each cell division until they activate DNA damage responses (DDR) and cell cycle inhibitors like p16 and p21, leading to senescence [13-15]. Genotoxic chemotherapy agents, including bleomycin and actinomycin D, induce DNA damage, while others, such as cisplatin and mitomycin C, interfere with DNA replication and transcription, thereby prompting senescence [16-34]. Oxidative stress and mitochondrial DNA mutations also contribute to senescence [35]. Mitochondrial dysfunction, indicated by reduced respiratory capacity and increased mitochondrial mass, is a hallmark of cellular aging [36]. Oxidative stress, resulting in high levels of reactive oxygen species (ROS), can damage mitochondrial DNA and promote aging [37-39]. Oncogene-induced senescence (OIS), such as overactivation of the Ras oncogene, represents another pathway leading to cellular aging [40-42].

At the core of cellular senescence lies cell cycle arrest, a primary contributor to the senescent state [43-45]. Stress-induced cellular senescence primarily involves two pathways: the p53-p21-DREAM-CDE/CHR pathway, where p53, a tumor suppressor, indirectly halts cell cycle progression and reduces gene expression, resulting in cell cycle arrest, apoptosis, or senescence [4, 46-49]; and the p16-CDK4/6-Rb pathway, where p16, an inhibitor of CDK4/6, regulates cell cycle arrest by influencing the phosphorylation state of the Rb protein [50, 51].

Cellular senescence is characterized by several features, most notably cell cycle arrest achieved by upregulating CDK inhibitors such as p21, p16 and p53, which have been widely used as biomarkers for detecting cellular senescence [1, 12]. Senescent cells also exhibit the senescence-associated secretory phenotype (SASP), involving the secretion of inflammatory cytokines, matrix metalloproteinases, microRNAs, chemokines, growth factors, and small molecule metabolites [43, 52, 53]. SASP is primarily mediated by NF-κB and regulated by transcription factors such as C/EBP-β and GATA4 [54, 55]. SASP includes numerous pro-inflammatory mediators, including IL-6, IL-8 and TNF-α, whose overproduction can trigger chronic inflammation and damage to adjacent tissues. However, due to its non-specific nature, SASP is not used as a standalone biomarker but can serve as an adjunct for validation [53, 56].

Senescent cells are also resistant to apoptosis, largely due to the upregulation of anti-apoptotic BCL-2 family members such as Bcl-2, Bcl-w and Bcl-xL [57, 58]. This resistance can be further strengthened by the chronic activation of the transcription factor CREB, which inhibits the downregulation of Bcl-2 expression [59]. Although these anti-apoptotic proteins are prominent in cellular senescence, they are not universally used as aging markers due to their expression in other contexts, such as non-senescent blood cells [60].

Morphologically, senescent cells often have increased cell volume and expansion due to mTOR pathway activation [61, 62]. At the organelle level, senescent cells typically have elevated levels of lysosomal SA-β-Gal enzyme, a lysosomal enzyme encoded by the GLB1 gene, which is optimal at a pH of 6.0 and serves as an effective biomarker for cellular senescence [1, 63-65]. Additionally, the accumulation of lipofuscin within lysosomes is indicative of aging [66]. Senescent cells display alterations in mitochondria and endoplasmic reticulum, including increased mitochondrial number and volume, distorted structures, and UPR-induced changes. Nuclear alterations are evident through telomere shortening and replication errors [67, 68], with significant chromatin reorganization due to DNA damage, resulting in senescence-associated heterochromatin foci (SAHF) [69]. The presence of γH2AX, a marker of DNA double-strand breaks, can be detected using various experimental techniques [70].

**Figure 1.**
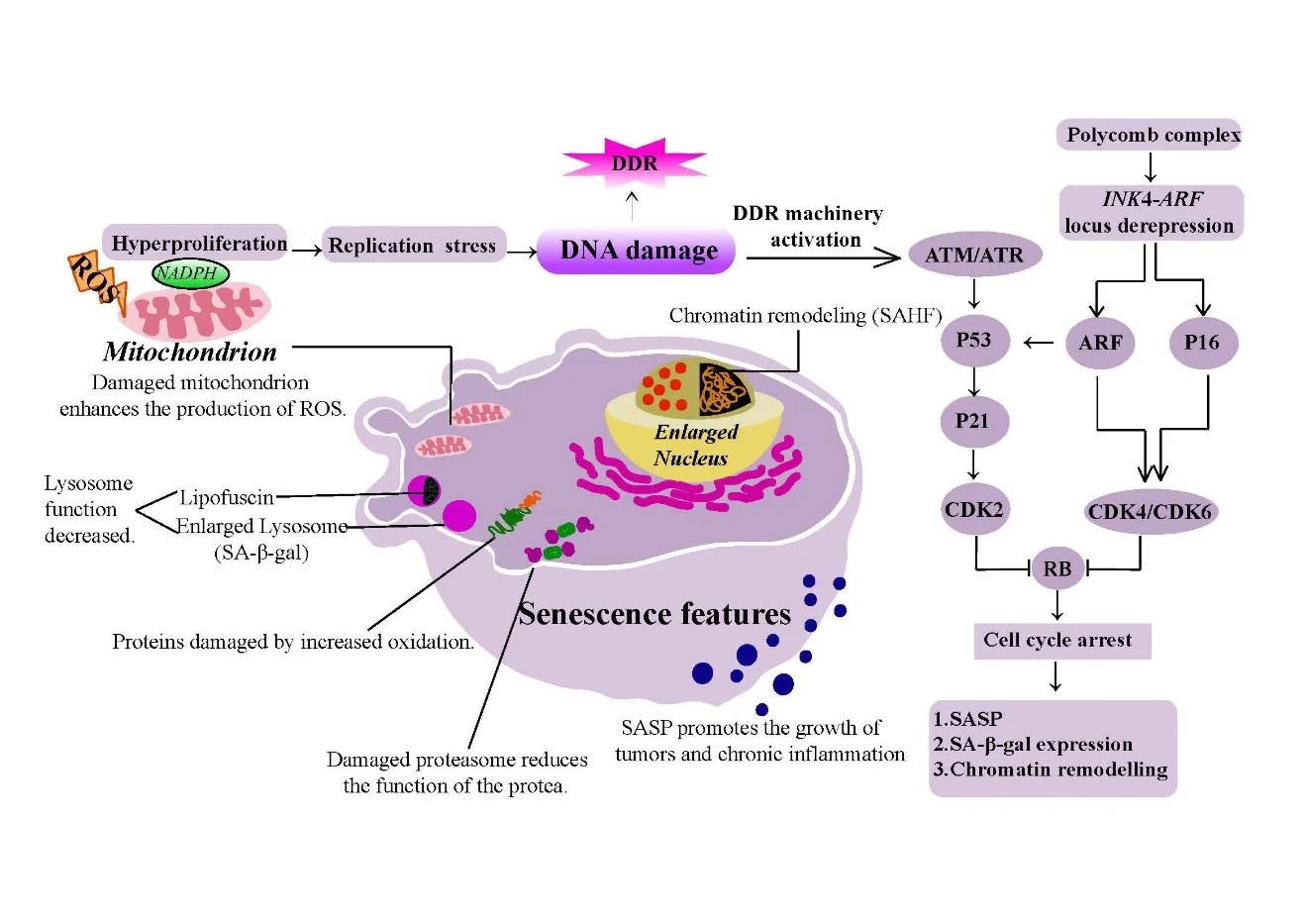
**Features and mechanisms of cellular senescence**. Senescence results from internal and external factors and is marked by stable growth arrest, SASP, DNA damage, and chromatin remodeling.

Taken together, cellular senescence is a complex process influenced by various intrinsic and extrinsic factors, including telomere attrition, DNA damage, mitochondrial dysfunction, and oncogene activation. The characteristics of senescence, such as cell cycle arrest, SASP, resistance to apoptosis, and alterations in cell and organelle morphology, signify the senescent state (Fig. 1). Understanding the mechanisms and hallmarks of cellular aging not only provides greater insights into its features but also assists in selecting appropriate markers and technologies for detecting senescence. Accurately identifying senescent cells is essential for advancing research on aging mechanisms, unraveling the intricacies of cellular aging and associated diseases, and developing interventions to delay aging and treat age-related conditions. The subsequent sections review important markers and techniques for detecting cellular senescence, serving as essential tools for enhancing our understanding and addressing the challenges of aging.

## 2. Conventional strategies

Numerous techniques exist for detecting senescence, including several classical and traditional experimental methods that have been used extensively over time, such as WB, qRT-PCR, ELISA, IHC, SA-β-galactosidase (SA-β-gal) staining, and Sudan Black B (SBB) staining, which remain the primary choices for detecting cellular senescence in mice. Of course, emerging technologies are also available. The first and foremost are the classic experimental techniques.

WB, also known as immunoblotting, is a widely used method for detecting and analyzing target proteins. Its invention was inspired by nucleic acid blotting [71]. In WB experiments, protein fractions with different molecular weights are separated by SDS-PAGE gel electrophoresis under the influence of an electric field, which transfers the proteins from the gel onto specific membranes (such as PVDF membrane, NC membrane, or nylon membrane) using the "sandwich" transfer membrane method, followed by the addition of "primary antibody" and "secondary antibody" for binding reaction. Lastly, the target protein is visualized through substrate color development or radiography.

In addition to WB, qPCR is another commonly used technical method for detecting biological indicators of aging in mice. Since the first fluorescent quantitative PCR detection system came out in 1996, PCR technology has undergone extensive development. The reaction process can now be visualized, allowing for real-time detection and quantitative analysis of results, enabling qPCR to be widely applied in various fields of life science research [72]. Commonly used methods of qPCR include fluorescent dye (SYBR Green I) and fluorescent probe (TaqMan Probe). The TaqMan probe is considered more specific than SYBR Green I. However, due to the ease of contamination in qPCR samples, some researchers believe that obtaining high-quality results with qPCR can be challenging [73]. In response to these concerns, the development of digital PCR (dPCR) may address the experimental needs of more researchers. Digital PCR not only avoids contamination issues but also enables the measurement of multiple gene targets with a single sample [74].

**Table 1:** Conventional detection methods.

Detection technique	Mouse strain	Sample category	Technical nature	Biomarkers	Ref.
**WB**	C57BL/6J	A protein sample of a cell or tissue	Qualitative and semi-quantitative	**Cycle protein arrest:** p53, Acetyl-p53, p21, Cdk2, Cdk4, Cdk6, NF-κB, p-NF-κB, Lamin B1, p62, p-p70S6K	[134-143]
				**Cell apoptosis block:** Cleaved caspase-3, Bax, FOXO1, FOXO3, FOXO4	
				**DNA damage:** γH2A.X, Histone H3, HMGB1	
				**Metabolic disorder:** MondoA, Rubicon, Prdx3, LC3, CPT1-α, ACADL, PPAR-α, PGC-1α, SIRT3, AMPK, cytochrome *C*, CypD, Nrf2	
				**SASP:** PAI-1, MMP3	
**qPCR**	C57BL/6J	RNA samples of cells or tissues	Qualitative and quantitative	**Cycle protein arrest:** Cdk2, Cdk4, Cdk6, p16, p21, p53, Ankrd1, Lamin B1	[134- 138, 140-147]
				**Cell apoptosis block:** Bax, Caspase-3, FOXO1, FOXO3, FOXO4, Bcl-2, Bcl-xl, Bcl-w	
				**DNA damage:** Tert, Runx1, GADD45a, FADS1, FADS2	
				**Metabolic disorder:** PPARα, PGC-1α, ACOX1, Trx1, Nrf2, SOD1	
				**SASP:** Cav1, IL-1β, IL-6, IL-8, Mmp3, Mmp9, Mmp12, PAI-1, TNF-α, Ccl2, Ccl4, Ccl-20, Ccl-7, TGF-β, CD31, Cd68, Cxcl1, Cxcl-3, Cxcl-10, Mcp1, GM-CSF	
**ELISA**	C57BL/6J	Blood, tissue/cell lysates	Qualitative and quantitative	**SASP:** IL-6, CXCL-1	[138, 140]
**IF Staining**	C57BL/6J	A section of cells, tissues or organs	Qualitative, positioning	**Cycle protein arrest:** P21, p53, p16 LaminB1, HMGB1, Pax7, pS6	[138, 140, 141, 148]
				**Cell apoptosis block:** BCL2, FOXO4	
				**DNA damage:** γH2A.X, 53BP1	
				**Metabolic disorder:** pAcGFP1-Mito, Hochest, RFP-LC3, MondoA, pMET, Tom20	
				**Other:** GFAP	
**IHC** **Staining**	C57BL/6J	A section of cells, tissues or organs	Qualitative, positioninng, relative quantification	**Cycle protein arrest:** P21, p16, Ki67, PCNA, pRb	[135, 136, 145, 148]
				**DNA damage:** γH2A.X	
				**Other:** CD66B	
**SA-β-gal Staining**	C57BL/6J	A section of cells, tissues or organs	Qualitative	β-galactosidase	[136-142, 147, 148]
**SBB** **Staining**	C57BL/6J	A section of cells, tissues or organs	Qualitative	β-galactosidase lipofuscinn	[135, 142]

ELISA relies on the specific binding of antigens and antibodies to detect and quantify molecules related to cellular senescence. ELISA was primarily developed in 1971 by Swiss scientists Engvall and Perlmann [75]. The method encompasses four types: the direct method, indirect method, sandwich method, and competition/ inhibition method. Typically, corresponding kits are used for experiments involving ELISA.

Immunohistochemistry (IHC) and Immuno-fluorescence (IF) are experimental techniques based on immunology, biochemistry and microscopy and are important for detecting senescent cells in mice. Since Coons et al. first used fluorescein to identify pneumococcus in 1942 [76], these techniques have undergone decades of refinement. Both IHC and IF methods rely on the specific binding between antigens and antibodies, utilizing various chromogenic agents to label cells for observation [77]. However, conventional IHC and IF techniques have limitations, including the ability to observe only one marker per tissue section, which poses challenges in specimen requirements. Presently, the advancement of multiple IHC/IF technology addresses these limitations. Multiplex IHC/IF technology facilitates high-throughput multiple staining and standardized quantitative analysis, propelling IHC/IF technology to a new height [78].

WB, qPCR and IHC are mainly used to detect cell cycle arrest related molecules, such as p53, p21, RB, and p16. They can also be utilized to detect molecules indicative of DNA damage. On the other hand, ELISA is mainly utilized for the detection of pro-inflammatory factors such as IL-6, IL-8, and TNF-α (Table 1).

Staining methods are often chosen to detect specific substances in senescent cells. For instance, SA-β-gal staining is a common method used to assess cell senescence [64]. In 1995, Dimri et al. reported the expression of β-galactosidase in human senescent fibroblasts, detectable at pH 6.0 [9]. Since then, SA-β-gal has been widely utilized as a biomarker for senescent cells in culture or mammalian tissues, effectively reflecting cellular senescence in vivo and in vitro [79]. The SA-β-gal staining method uses β-galactosidase to hydrolyze the X-gal substrate at pH 6.0, producing a dark blue color in senescent cells, which is observable with a light microscope.

SBB staining is a recognized histological technique utilized for the detection of lipofuscin, a pigment that was initially characterized by Sheehan in 1939 for the staining of white blood cells [80]. Subsequent research has elucidated that lipofuscin accumulates within cells as they age, thereby serving as an indicator of cellular senescence [81]. As a lipophilic dye, SBB uniquely binds to lipofuscin, allowing for its visualization under a light microscope [82]. This staining method has since become a valuable tool in the study of aging and cellular degeneration processes. Compared to SA-β-gal staining, which is limited to fresh tissues and requires rapid freezing, SBB is more widely used, ensuring the accuracy of aging cell detection [66, 83].

In general, WB and qPCR offer molecular insights into the degree of cellular senescence in mice, while microscopic techniques such as IF and IHC provide detailed cellular-level observations that are intuitive and clear. Each method has its strengths and limitations, as outlined in Table 2. The choice of technique often depends on the specific requirements of the study at hand. The collective use of these conventional strategies remains a cornerstone in the comprehensive assessment of cellular senescence.

## 3. Novel strategies

### 3.1 Gene-edited reporter mice

Mice are important for studying mammalian development due to their close genetic similarity to humans and several advantages. With the advancement of genetic tools, scientists can now precisely manipulate the animal genome. Reporter mice are a direct result of these genetic tools. Following the generation of the transgenic Green mouse, which expresses GFP, in 1997 [84], fluorescent proteins have become more widely utilized for visualizing proteins of interest and the dynamic aspects of gene expression and cell signaling pathways. The fundamental technology behind reporter mice is the reporter gene. Compared to traditional approaches, the use of reporter mice offers numerous advantages. Unlike methods that require the sacrifice of mice to obtain tissue samples, reporter mice provide a non-invasive means of real-time monitoring and visualization [85, 86]. Since traditional methods often involve sampling after cell activity has ceased, some dynamic processes in cell activities are challenging to explore. Reporter mice enable the detection of live cell activities in vivo in real-time, such as tracking specific molecules in cell signaling pathways. Moreover, the application of reporter mice allows the detection to be retained in vivo, making the experimental process non-toxic and easy to observe. Reporter genes can be utilized to detect gene expression and promoter activity [87, 88]. Combined with their performance in sensitivity, dynamic range, convenience and reliability [89-91], reporter mice are increasingly used for detecting cellular senescence.

**Table 2:** Advantages and disadvantages of conventional methods.

	WB	qPCR	SA-β-gal	IF	IHC	SBB
**Advantages**	High specificity and high sensitivity. One experiment can analyze multiple samples and a variety of indicators.	It can be quantitatively analyzed, the operation steps are simple, the sensitivity is high, the specificity is strong (TaqMan method is stronger), the repeatability is good, the sample volume is small, the fluorescent dye method is relatively cheap, and more.	The method is simple, highly specific, intuitive and reliable, has a wide range of applications (cell culture in vitro and tissue sections in vivo can be used, and living tissues can also be used), and the cost of consumables is low.	It has high specificity, high sensitivity, fast speed, no radioactive contamination, and simple operation steps.	It has high specificity, high sensitivity, accurate positioning and easy sample storage.	Simple operation, economical, high sensitivity, easy visualization and high stability (stained samples can be stored for a long time).
**Disadvantages**	Long operation time. The operation steps are complicated, and mistakes are common. Antibody consumables are more expensive.	The design of primers and probes is demanding, the experimental environment is demanding, pollution prevention is necessary, the whole process of ice operation (RNA extraction is easily contaminated by ribonuclease and degrades the sample), the most error-prone when adding samples (many times of loading, changing the tip one by one, and it is difficult to control the generation of bubbles), and the specificity of fluorescent dye method is lower than that of fluorescent probe method. The probe is expensive to synthesize.	The results of observation and analysis are highly subjective, can only be used for qualitative judgment of aging, and can only indirectly reflect the state of aging, but can not directly reflect the molecular changes.	Non-specific staining, result judgment is not objective, technical process is complex, and quantitative measurement is difficult.	The operation is complex, and the repeatability is low	Non-specific staining, high background and low contrast, and difficulty in quantitative analysis.

Over the past decade, significant progress has been made in the development of various reporter mice used to detect cell senescence. Here, we summarize the reporter mouse models that have been applied for detecting cell senescence in the past 15 years.

Due to the fact that there is no absolutely specific biomarker for senescent cells, different biomarkers are used for different models when detecting senescent cells in mice. p16, a protein involved in cell cycle regulation, has been widely recognized as a marker of cellular senescence. Consequently, numerous reporter mice have been designed for p16 detection. The initial two types of reporter mice utilized luciferase. These mice do not necessitate external light excitation but require luciferin as a consumable substrate. Typically, fluorescent substrates need to be administered to mice via intraperitoneal injection several minutes before imaging. The two mouse strains employed distinctly different gene editing methods. Dr. Hara's group generated the mouse transgenically using pronuclear microinjection of the BAC vector into fertilized oocytes [92], while Dr. Sharpless's group employed a targeted "knock-in" strategy [93]. Another type of reporter mouse, the p16^tdTom/+^ mouse, was developed using a "knock-in" strategy by Dr. Sharpless's group in 2019 [94]. In this model, the reporter gene encoding tdTomato, a non-toxic and highly fluorescent protein with strong tissue penetration as a red fluorescent marker, was inserted into exon 1α of the p16^Ink4a^ gene. In 2011, Dr. van Deursen's group created the INK-ATTAC mouse model, which is capable of monitoring and eliminating senescent cells simultaneously [95]. Following a similar construction principle to other models, INK-ATTAC incorporated an EGFP gene following an internal ribosome entry (IRES) to the 2617-bp fragment of the p16^Ink4a^ gene promoter, allowing senescent cells to express the EGFP reporter protein. Additionally, an FKBP-Casp8 fragment was introduced under the same promoter. The protein encoded by the fragment can be dimerized upon induction by the drug AP20187, thus clearing senescent cells. The third reporter mouse model targeting p16^Ink4a^ is the p16-3MR mouse, developed by Dr. Campisi's group in 2014 [96]. The 3MR fusion protein comprises synthetic Renilla luciferase (LUC), monomeric red fluorescent protein (mRFP), and truncated herpes simplex virus 1 (HSV-1) domains, all under the control of the p16 promoter [96]. Notably, both luciferase and fluorescent protein were utilized in this model, where LUC facilitates the detection of cells expressing 3MR, and mRFP allows for the selection of senescent cells from tissues. Similar to the mechanism in INK-ATTAC, senescent cells expressing HSV-TK can be cleared by the drug ganciclovir (GCV) due to GCV's higher affinity for HSV-TK than cellular TK. This results in mitochondrial DNA fragmentation and cell death via apoptosis [97]. While the aforementioned animal models have garnered considerable use, they encounter specific challenges that cannot be overlooked. For example, the INK-ATTAC system has demonstrated inefficiencies in the removal of p16-expressing cells in various tissues, such as the liver, colon, and T lymphocytes [98]. Furthermore, attempts to incorporate a fluorescent reporter into the targeting cassette have been rendered impractical due to the low levels of p16 mRNA expression in vivo [96]. To counteract these limitations, researchers have employed a reporter mouse model targeting p16^high^ expressing cells to delve into the in vivo dynamics and characteristics of these cells. Omori et al. generated p16^Ink4a^-Cre^ERT2^neo mice by replacing the first exon of the endogenous p16 ^Ink4a^ gene with a cassette containing a target gene [99]. In this model, tamoxifen (TAM) was administered to control Cre activity, enabling long-term labeling of cell proliferation and the determination of cell half-life. Subsequently, these mice were crossed with Rosa26-CAG-lsl-tdTomato mice to produce p16^Ink4a^ -Cre^ERT2^ neo-tdTomato mice, which are capable of specifically labeling p16^high^ expressing cells upon TAM administration. The reporter mice can track and label p16^high^ cells in real-time. And the hybridization step used to generate this mouse ensures specific labeling of cells with high p16 expression, thereby minimizing false positive signals. Another series of reporter mice were p16-Cre/R26-mTmG and p16-Cre/R26-DTA designed by Grosse et al [100] . First, the team generated p16-Cre mice by gene insertion, Southern blot selection, and breeding. Subsequently, p16-Cre mice were crossed with other reporter mice to obtain specific reporter strain. Mice bred with Rosa26-mT/mG will continuously mark p16-expressing cells, while those bred with Rosa26-DTA will selectively eliminate these cells by leveraging the toxicity of DTA when it is released from the cells. The mT/mG effect results in the expression of red fluorescence from tdTomato in the absence of Cre and green fluorescence of EGFP in the presence of Cre exposure. In brief, these two reporter mice can monitor and eliminate p16-expressing cells through the action of Cre.

While p16 is commonly used as a biomarker for senescent cells, not all senescent cells express high levels of p16, and elevated p16 levels do not necessarily indicate cellular senescence [12]. Another widely utilized senescent cell marker is p21, with an increasing number of studies highlighting its significance in aging-related diseases [101-103]. This review focuses on three types of reporter mouse models designed for p21 detection. In 2021, Dr. Xu's group developed a p21-Cre mouse model capable of monitoring and manipulating p21-highly-expressing senescent cells in vivo [104]. This model incorporates several sequences under the control of the p21 promoter. One sequence encodes a fusion protein of Cre recombinase (Cre) fused to a tamoxifen-inducible estrogen receptor (ERT2) domain, while another sequence contains an IRES followed by an open reading frame (ORF) encoding enhanced GFP, facilitating the sorting and detection of cells. Ingeniously, the p21-Cre mouse model offers an indirect approach to monitoring, sorting, imaging, eliminating, or modulating p21-high-expressing cells by crossing the model with floxed mice [104]. Since ERT2 translocates from the cytoplasm to the nucleus to act on the loxP site preferentially when induced by tamoxifen or 4-hydroxytamoxifen, Cre activity can be regulated based on the presence or absence of the inducer. The initial cross, p21-Cre-/+; LUC/+(PL) mice, involved mating p21-Cre mice with floxed knock-in LUC mice, which contain a loxP-flanked STOP fragment between the Gt (ROSA)26Sor (ROSA) promoter and LUC [105]. Thus, proper Cre activity enables the expression of LUC, facilitating bioluminescence imaging in live mice. Additionally, the chemotherapeutic drug doxorubicin (DOXO) functions as a DNA-damaging agent to induce p21 expression [106]. Therefore, high LUC activity in DOXO-treated PL mice signifies functional transgene activity. The subsequent cross, termed PT mice, involves mating p21-Cre mice with floxed knock-in tdTomato mice [107]. Unlike PL mice, PT mice utilize the CMV early enhancer/chicken β-actin (CAG) promoter and the tdTomato reporter gene in the floxed mice crossed with p21-Cre mice. The robust CAG promoter and the red fluorescent protein tdTomato render PT mice suitable for in vivo fluorescent imaging. Another cross is between the initial cross (PL mice) and floxed diphtheria toxin (DTA) mice [108]. In comparison to PL mice, the derived p21-Cre/+; LUC/DTA (PLD) mice can express DTA with the aid of Cre, inducing apoptosis in p21-expressing senescent cells. Utilizing PL mice for this cross allows researchers to validate clearance by comparing LUC activity between PL mice and PLD mice. The final cross involves mating p21-Cre mice with floxed Rela mice [109]. In this model, Cre-mediated inactivation of the NF-κB pathway alleviates the harmful effects of SASP by genetic inhibition, demonstrating the modulating function of the mouse model. Overall, this p21-Cre mouse model serves as a multifunctional system capable of monitoring, modulating, imaging, and more. Another mouse model is the p21 version of the 3MR reporter mouse developed by Yi et al. in 2023 [110]. The 3MR transgene was knocked in via CRISPR-Cas9 technology instead of using a BAC vector [110]. Comparatively, the p21-3MR mouse additionally validates transgene activity compared to its p16 counterpart with the assistance of DOXO. Lastly, the p21-Fluc mice, generated by Tinkum et al., have luciferase labeling the p21 endogenous promoter and accurately report p21 expression [106].

p53 plays important roles in regulating cell cycle arrest, apoptosis and genome stability through various mechanisms. To monitor p53 activity both in vivo and in vitro, Goh et al. generated two reporter mice: p21p53RE-EGFP and Pumap53RE-EGFP [111]. In these mice, EGFP expression is driven by p53 transcriptional activity at response elements from the p21/Puma promoter [111]. This setup enabled the detection of changes in p53 activity in response to different stimuli, tissue types, and response elements. Such models prove invaluable for drug research in cancer and aging.

SASP is one of the hallmarks of cellular senescence, which involves the secretion of numerous factors, indicating the involvement of multiple molecules. Among these, the NF-κB pathway is a key regulator of SASP [112]. Sung's team developed NF-κB double knock-in reporter mice using CRISPR-Cas9 technology [113]. It is noteworthy that the reporter molecules are linked not to the entire biomarker but respectively to its two subunits: mEGFP attached to RelA and mScarlet attached to c-Rel. Interestingly, during physiological aging, microglia subpopulations tend to shift towards c-Rel-driven amplification of NF-κB signaling [113]. This method of labeling subunits offers insights into the coordination of subunits in signal transduction and proves useful for studying complex signaling pathways like SASP.

In 2021, Liu et al. introduced the Glb1-2A-mCherry reporter mice, building upon the foundation of SA-β-gal staining, a hallmark indicating an increase in lysosomal mass and β-galactosidase (β-gal) protein [114, 115]. Sequences encoding mCherry were incorporated at the 3' end of the Glb1 gene, responsible for encoding lysosomal β-D-galactosidase. This innovative reporter mouse offers a novel biomarker distinct from the traditional senescence markers p21 and p16, thus providing a valuable addition to the repertoire of senescence detection methods. Given the limitations of individual biomarkers in capturing all forms of cellular senescence [12], the utilization of Glb1 represents a significant completion in this field.

Overall, the emergence of gene-edited reporter mice has not fundamentally altered the selection of biomarkers for detecting senescence. Instead, it has introduced a dynamic, real-time platform that mitigates the impact of sampling processes. This technological advancement has paved the way for innovative research methodologies, leading to a more profound and detailed comprehension of cellular senescence and its implications in aging and disease. A comprehensive overview of the reporter mice discussed is shown in Table 3.

### 3.2 Probes

A probe refers to a specially designed molecule engineered to interact with specific analytes, such as senescent markers, thereby inducing measurable changes in properties. Probe technology offers a sensitive and efficient approach to detect cellular senescence. While gene-edited mice are commonly utilized for this purpose, probes predominantly target β-galactosidase due to its enzyme properties that align well with probe compatibility. Additionally, other probes have been developed for senescence detection.

**Table 3:** Novel detection methods.

Biomarker	Mouse strain	Reporter mice	Reporter	Techniques	Application	Ref.
**p16**	C57BL/6	p16-3MR mice	Luciferase, mRFP, HSV-TK	Transgenic	To detect, sort and kill p16-positive cells	[96]
**p16**	C57BL/6	p16^tdTom/+^ mice	tdTomato	Knock-in	To detect and isolate individual senescent cells	[94]
**p16**	ICR	p16-LUC	Luciferase	Transgenic	Make real-time imaging of senescent cells with LUC	[92]
**p16**	-	p16-LUC	luciferase	Knock-in	Make real-time imaging of senescent cells with LUC	[93]
**p16**	FVB	INK-ATTAC reporter mice	GFP, ATTAC	Transgenic	To detect, isolate and kill senescent cells	[95]
**p16**	C57BL/6	p16^Ink4a^-Cre^ERT2^neo-tdTomato mice	tdTomato	knockin	To detect senescent cells in real-time	[99]
**p16**	-	p16-Cre/R26-mTmG & p16-Cre/R26-DTA	tdTomato, EGFP	knockin	To detect and kill senescent cells	[100]
**p21**	C57BL/6	p21-Cre	GFP, luciferase, tdTomato	Knock-in	To monitor, regulate and clear senescent cells by crossing p21-Cre mice to floxed mice	[104]
**p21**	C57BL/6	p21-3MR mice	Luciferase, mRFP, HSV-TK	Transgenic	To detect, sort and kill p21-positive cells	[110]
**p21**	-	p21-LUC	Luciferase	Knock-in	Make real-time imaging of senescent cells with LUC	[106]
**p53**	C57BL/6	p21p53RE-EGFP & Pumap53RE-EGFP	EGFP	Transgenic	To monitor p53 activity, detect variations in p53 activity according to response element, tissue type, and stimulus	[111]
**NF-κB (RelA & c-Rel)**	C57BL/6	NF-κB double knock-in reporter mice	mEGFP, mScarlet	Knock-in	To study spatiotemporal dynamics of NF-κB	[113]
**Glb1**	C57BL/6	Glb1-2A-mCherry reporter mice	mCherry	Knock-in	To make real-time monitoring of systemic aging and organ function decline	[115]

#### 3.2.1 β-gal Probes

Unlike gene-edited mice, most probes are designed to target β-gal for senescence detection. Various imaging modalities are employed, including fluorescence imaging, bioluminescence (BL) imaging, chemiluminescence (CL) imaging, and photoacoustic (PA) imaging. Photoacoustic (PA) imaging is a novel technique that combines the advantages of optical and ultrasound imaging. It directs light to the substance produced in the reaction, and the ultrasound transducer detects the resulting ultrasound wave, offering high spatial resolution and deep tissue penetration. In the minority are bioluminescence and chemiluminescence. CL involves light emission resulting from chemical reactions. BL does not require excitation light; the probe illuminates when oxyluciferin's chemical energy converts to light energy Designed by Blau, probes spatially restrict Fluc for luciferin catalysis initially, releasing luciferin upon reaction with β-gal, which then luminesces in the presence of ATP, Mg^2+^, and O_2_. Most probes are fluorescent, with fluorogenic substrates fluorescing upon excitation by external light source [116]. Relatively, it is an advantage for fluorescent probes to choose appropriate wavelengths of light with strong penetrating power like NIR. Fluorescent probes employ two detection approaches: Turn-On and ratiometric. Turn-On probes, when reacting with β-gal, hydrolyze the glycosidic bond, restoring fluorescence signal. Ratiometric probes rely on the ICT effect, where molecules exhibit bathochromic (red) or hypsochromic (blue) shifts in absorption and emission spectra, enabling analyte positivity indication via fluorescence intensity ratios from two different wavelengths. Furthermore, based on fluorescence, a series of two-photon fluorescence (TPF) probes were designed, utilizing two NIR photons to achieve lower autofluorescence and photodamage [117-120].

Several reviews have explored probes targeting β-gal. Feng et al. [121] and Yao et al. [122] have summarized fluorescent probes in their respective works. Zhang et al. [123] have categorized probes based on their luminescence mechanisms, including fluorescent, bioluminescent (BL), chemiluminescent (CL), and photoacoustic (PA) probes. Lozano-Torres et al. [124] have provided a comprehensive overview of probes based on their applicability in solutions and in vitro or in vivo settings. This review complements existing literature by introducing two novel CL probes.

In terms of CL probes, Xu et al. made a significant advancement with the creation of HPQCL-Cl. This probe combines Schaap's dioxetane with an ordered-assembly HPQ dye driven by hydrogen bonding, and the resulting HPQCL-Cl-β-gal probe enables long-term imaging with a high signal-to-noise ratio, showing promise for guiding clinical surgery due to its prolonged half-life [125]. Meanwhile, Tennous et al. focused on optimizing the chemiexcitation rate of phenoxy-1,2-dioxetane luminophores by incorporating spirostrain released in the decomposition of 1,2-dioxetane luminophores [126], leading to increased detection sensitivity and achieving the highest signal-to-noise ratio.

#### 3.2.2 Sialidase probe

Zhu et al. introduced a novel probe called Sia-RQ (λex=580 nm) designed to label the emerging senescent biomarker α2-3,6,8 neuraminidase (sialidase) [127]. The probe includes a Sia entity recognized by sialidase. Upon desialylation, Sia-RQ self-immolates, releasing the blackhole fluorescence quencher (BHQ) and thus restoring the rhodamine-X fluorophore paired with BHQ. This probe enables wash-free imaging of senescence-associated sialidase in vitro but not in vivo. The use of sialidase as a biomarker offers a complementary approach to the prevalent use of β-gal as a probe target.

#### 3.2.3 ROS probe

Narayanaswamy et al. introduced an NIR probe QCy-BA designed to detect H_2_O_2_ produced by EGF/Nox pathways and post-genotoxic stress in both normal and senescent cells [128]. H_2_O_2_ was chosen as the target due to its prominence among ROS [129, 130] as well as QCy-BA's high selectivity for it. The QCy-BA reacts with H_2_O_2_, releasing the sequence-specific DNA minor groove probe QCy-DT, which then exhibits turn-on NIR fluorescence when combined with AT-rich DNA. Importantly, the reaction between H_2_O_2_ and boronic acid or ester on the probe is chemo-specific, bio-orthogonal and biocompatible, with non-toxic byproducts for living cells [128]. Although it is a fact that senescent cells generally exhibit higher ROS levels than normal cells [129], high level of ROS alone is not a specific indicator of senescence. However, QCy-BA can serve as a supplementary indicator, providing a reference for more robust methods.

In summary, probe technology offers a sensitive and convenient approach for molecular assays, enabling real-time monitoring of biological processes. The diverse range of available probes provides numerous methods for detection and monitoring, and as this technology advances, it is expected to offer even more potent tools for studying cellular senescence and related biological phenomena.

## 4. Comparison of conventional strategies and novel strategies

Traditional methodologies frequently encounter challenges such as being cumbersome and time-consuming, which can significantly hinder operational accuracy and efficiency. These approaches are often labor-intensive and do not offer the immediacy of real-time data, which is essential for effective research and decision-making. Furthermore, the detection of cellular senescence and its biomarkers is complicated by the inherent diversity and complexity across different organisms and even within various tissues of the same species. The current techniques, while valuable, are often cumbersome and susceptible to operational and environmental factors, which can compromise their precision and flexibility. This lack of adaptability is particularly problematic in the context of diverse research settings where the need for accurate and reliable detection is paramount.

The emergence of novel strategies has transformed the observation of senescent cells compared to conventional methods. Through the use of gene-edited mice or specialized probes, scientists can now monitor dynamic biomarkers and biological processes in real time, marking a significant advancement over traditional approaches. This innovative approach not only reduces the time and number of mice required but also provides a distinct advantage in aging studies, where physiological parameters can fluctuate over time or in response to treatments. Furthermore, by avoiding the need to sacrifice animals, this approach eliminates the potential introduction of unpredictable changes that can occur during sample collection and processing, thereby reducing errors. In vivo monitoring offers a more accurate reflection of the natural physiological state, thereby enhancing the representativeness of the results. Additionally, these innovative technologies offer greater flexibility in experimental design, simplifying the process and expediting drug discovery efforts.

While gene-edited mice offer significant advantages, they also pose challenges. The process of creating reporter mice is complex and multi-step: it involves selecting the target gene and promoter, constructing the vector, introducing it into embryonic stem cells, generating chimeric mice, and then breeding and identifying the desired offspring. It seems that the quickest way to acquire report mice is to purchase pre-made reporter mice from specialized agencies. However, the high costs of using these mice, including the expense of the animals themselves and compatible detection equipment, are significant considerations. To mitigate these challenges, fostering collaborative networks among research institutions is a strategic approach. By establishing shared resources, such as a communal mouse colony, and implementing a system of resource pooling, the financial burden can be more equitably distributed. This collaborative spirit can lead to more efficient use of resources and reduce costs for individuals. Moreover, the dynamic nature of target biomarkers necessitates the creation of numerous reporter mice, which is a labor-intensive and costly process compared to the simple adjustments required for conventional molecular assays, such as changing antibodies or primers. To streamline this process, leveraging bioinformatics tools and computational models is essential. These tools can predict the behavior of senescence biomarkers and simulate the effects of different reporter mice strains, thereby reducing the necessity for extensive physical testing.

In contrast, probes provide a more flexible alternative to gene-edited mice. Their lightweight and adaptable design enables their use in various settings, including in vivo, in vitro, and in solution. The flexibility to interchange recognition units allows probes to be easily repurposed for detecting various biomarkers, unlike the creation of new mouse strains, which is complex and time-consuming. However, using probe technology to detect biomarkers requires meticulous data processing. This involves carefully managing background signals with blocking agents and optimal washing conditions, precisely optimizing hybridization conditions such as temperature and salt concentration and employing rigorous control samples to evaluate non-specific signals and ensure experimental consistency. Thorough data correction and analysis are needed, including subtracting background noise and using standard curves for quantification. Additionally, results must be validated through repeated experiments and cross-verification with alternative methods. As a result, comprehensive recording, management, and reporting of all experimental data are essential to ensure clarity and reproducibility.

In summary, while traditional methods have their strengths, the newer approaches using gene-edited mice and probes represent a significant advancement in cellular aging research. These innovative techniques improve study efficiency and precision while also offering greater flexibility in experimental design. Gene-edited mice, with their technological sophistication, offer detailed insights into the aging process, while probes, known for their cost-effectiveness, deliver reliable and accurate results. Combining new technologies with traditional ones could ensure their wide application in various research contexts. For example, in establishing a reporter mouse model, traditional molecular biology methods can verify the successful implantation of the target gene. Additionally, since senescent cells exhibit a variety of aging markers, traditional molecular biology techniques can provide robust validation for potential new markers due to their reliability. This dual approach not only accelerates the discovery process but also strengthens the validation of findings. However, to harness the full potential of these cutting-edge techniques, it is essential to address challenges such as cost and data analysis complexity to fully utilize the potential of these innovative techniques in advancing our understanding of cellular aging and developing effective therapeutic interventions.

## Conclusion and prospect

As technology and society evolve, there is an increased focus on understanding and addressing the complexities of aging. Aging research has become increasingly pivotal, aiming to uncover the nuances of the aging process. Over the past decade, researchers have not only continued to use traditional methods for detecting signs of aging but have also innovated by developing novel techniques to enhance efficiency and comprehensiveness in detection. This review synthesized the landscape of traditional cell senescence detection methods and highlights groundbreaking new technologies, such as the use of gene-edited mice and sophisticated probes.

Exploring strategies to detect cellular senescence is essential for enhancing our understanding of aging mechanisms, which can facilitate early prevention and intervention and allow progress for novel therapeutic approaches. A key advantage of these new strategies is their ability to dynamically detect senescence in real time, representing a paradigm shift in aging research. Moreover, the potential integration of these innovative technologies with other advancements, as demonstrated by the p21-Cre mouse model, opens up unprecedented possibilities for detecting, targeting, and even treating senescent cells in previously unexplored ways.

Despite methodological advances, existing methods still have shortcomings in certain research scenarios. For instance, there are issues with non-specific activation of biomarkers, limited time resolution due to insufficient signal accumulation in transient aging events and limited spatial resolution when detecting signals from deep within tissue. Ongoing efforts to refine these new technologies, along with advances in fluorescent materials and the discovery of novel chemiluminescence reaction substrates, hold promises for enhancing the effectiveness and broadening the application of these detection methods. However, it is important to acknowledge that no single, definitive biomarker of aging exists, such as the SASP, which can also be observed during chronic inflammation and immune system activation [52, 131]; markers like p21 and p16, which are intimately associated with tumorigenesis and tumor progression, are not exclusively indicative of senescence, as they are also implicated in cellular apoptosis [132, 133]. Consequently, detecting cell senescence necessitates a multifaceted approach that employs a combination of biomarkers to enhance specificity and sensitivity. In the realm of experimental techniques, it is imperative to integrate multidimensional detection methods with a judicious selection of biomarkers that can discern the nuanced manifestations of cellular aging.

In conclusion, the quest to understand and combat aging involves a multifaceted approach that depends on the continuous progress achieved in detection technologies. By using the potential of gene-edited mice, probes and other emerging tools, alongside ongoing efforts to identify new biomarkers and refine existing ones, researchers can make significant advancements in the study of aging. This progress not only deepens our understanding of the aging process but also holds promise for the development of effective interventions and therapies, ultimately improving the quality of life for aging individuals.
